# Direct and indirect effects of economic sanctions on health: a systematic narrative literature review

**DOI:** 10.1186/s12889-024-19750-w

**Published:** 2024-08-17

**Authors:** Vahid Yazdi-Feyzabadi, Atefeh Zolfagharnasab, Soheila Naghavi, Anahita Behzadi, Maysam Yousefi, Mohammad Bazyar

**Affiliations:** 1 Health Services Management Research Center, Institute for Futures Studies in Health, Kerman University of Medical Sciences, Kerman City, Iran; 2 Faculty of Management and Medical Information Sciences, Kerman University of Medical Sciences, Kerman City, Iran; 3 Health Services Management Research Center, Institute for Futures Studies in Health, Kerman University of Medical Sciences, Kerman City, Iran; 4 Research Center of Tropical and Infectious Diseases, Kerman University of medical sciences, Kerman, Iran; 5 Faculty of Health, Health Management and Economics Department, Ilam University of Medical Sciences, Ilam City, Iran

**Keywords:** Economic sanctions, Social determinants of health, Health system, Systematic review

## Abstract

**Background:**

Economic sanctions are defined as restrictions imposed by other countries against individuals, groups, or governments of other countries. These sanctions have a detrimental impact on the economies of countries and can also limit access to healthcare services for people as a secondary consequence. This study aims to systematically review the literature to examine the direct and indirect effects of economic sanctions on health through a narrative synthesis.

**Methods:**

This systematic literature review was limited to papers published between January 1990 and July 2023. Relevant documents published in English and Persian were searched for in databases including Cochrane Library, PubMed, Embase, Scopus, Web of Science, SID, Magiran, and Irandoc. The direct and indirect effects of sanctions on health were classified using two frameworks proposed by the World Health Organization (WHO): the Health System Building Blocks and “Social Determinants of Health”.

**Results:**

Out of a total of 18,219 articles, 59 were selected based on inclusion criteria. The effects of sanctions were divided into direct and indirect groups. Direct effects encompassed seven main themes: access to essential medicine, medical products, vaccines and technologies; financing; health workforce; service delivery; research and health information systems; health outcomes; and financial risk protection. Indirect effects also were classified into six main themes: socioeconomic status; food and agricultural products; stress; early life conditions; high-risk behaviors and addiction; and transport. Most studies focused on the access to medicines, food, economic and social status.

**Conclusions:**

Economic sanctions have had profoundly negative impacts on all aspects of the healthcare system. The international community must address these effects on health and take necessary measures to prevent or mitigate them, particularly in ensuring the provision of basic and essential healthcare needs for individuals and communities.

**Supplementary Information:**

The online version contains supplementary material available at 10.1186/s12889-024-19750-w.

## Background

Sanctions are purposeful and determined restrictions imposed by one or more countries against another individual, group or countries’ government. Sanctions are usually imposed by international organizations as a pressure tool for responding to the course of actions of any country that opposes them [1].

Economic sanctions are the most common type of these restrictions. The two main types of these sanctions are trade and financial restrictions. Trade sanctions restrict imports to and exports from the countries under sanctions while financial sanctions are closely related to economic ones, but their focus is on banning the money flows and financial resources into or out of the country. These sanctions can include blocking government assets, restricting access to financial markets, loans and credits limitations, restricting international financial exchange, and also sales and trade abroad [2].

Economic sanctions reduce people’s access to basic necessities of life by debilitating the economic situation, decreasing welfare and weakening the functions of the target country’s social systems. One of the most important areas affected through these boycotts is health. Due to the expansion of health scope, these limitations affect different parts of health system itself and as a result endanger people’s life [3, 4].

Studies in various countries, including Iran, Iraq, Cuba, Yugoslavia and Haiti, discussed the effects of sanctions on health. In Haiti, economic sanctions have reduced incomes, increased unemployment and poverty along with mortality by 1 to 4 years, and destroyed families [5]. In Iran, especially in healthcare area, sanctions have resulted in increasing the cost of essential procedures and drugs such as diagnostic procedures for cancers and chemotherapy drugs. The difficulties in getting required licenses for financial transactions and transportation insurance due to sanctions has left the country with a shortage of drugs and health equipment [6].

Sanctions have devastating effects on the health of vulnerable patients or health systems customers too. Patients who are suffering from diseases such as asthma, thalassemia, hemophilia, chronic diseases, blood disorders, multiple sclerosis and HIV/AIDS have limited access to drugs [7]. While comparing, in developed countries mortality rates decreased using appropriate drugs [8, 9].

Different countries may use broad policies to prevent or adjust the negative effects of economic problems on health systems, although these policies may not be successful in ensuring continued access to health services [10, 11].

Although sanctions may be designed for excluding medical products from the list, they can still have an inevitable impact on access to health services. Thus, the imposition of economic sanctions can threaten public health directly [12].

Furthermore, economic sanctions suppress the health indirectly by adversely impacting on other related parts known as social determinants of health (SDH) and Sustainable Development Goals (SDGs). Economic sanctions impact all aspects of the social determinants of health (SDH) framework, leading to negative effects on health equity and well-being. Sanctions can alter social and political systems, such as governance, labor markets, education, trade, housing, and redistributive policies, influencing people’s health. Structural determinants like income, education, and occupation are affected by sanctions, changing health opportunities and status, especially for the economically disadvantaged. Intermediary determinants, including material and psychosocial circumstances, are also influenced negatively by sanctions. For instance, housing quality declines post-sanctions due to increased costs of land and materials, while food consumption patterns shift towards cheaper, less nutritious options. Sanctions create psychosocial stressors like job insecurity and uncertainty, leading to frustration and stress [13–16].

Continued sanctions may hinder countries’ progress towards achieving Sustainable Development Goals (SDGs), particularly SDG-3 for healthy lives and well-being. Economic stability is crucial for meeting health-related SDGs, and any failures in this regard would disproportionately impact citizens in targeted countries. In the context of developing countries, where progress toward SDGs is often hindered by limited resources and systemic disparities, the impact of economic sanctions on health systems and overall well-being is profound. SDGs, with their emphasis on health (Goal 3) and the overarching aim of leaving no one behind, seek to address disparities and ensure equitable access to healthcare services. Economic sanctions, however, disrupt this delicate balance, exacerbating existing inequalities and impeding the ability of nations to meet the health-related targets outlined in the SDGs [13].

Given that, the effects of sanctions depend on the situation of countries and vary from one to another, there is no complete evidence of a comprehensive impact of sanctions on different part of society’s system especially in health despite of its importance. To comprehensively address these issues, a rigorous examination of evidence through narrative systematic reviews becomes imperative. This study aims to provide a detailed narrative synthesis of the direct and indirect effects of economic sanctions on health system building blocks and public health focusing on social determinants of health, thereby contributing to a better understanding of the broader consequences of such measures.

## Methods

The following steps were taken to review literature systematically [17, 18].

### Research question

The main question that we wanted to answer in this study was to investigate and categorize the effects that economic sanctions impose on health directly and indirectly.

### Search strategy and identifying literature

This systematic review was carried out according to the latest version of PRISMA guidelines [19, 20]. For the purposes of the study, following databases were searched by one of the authors experienced in systematic research: Cochrane Library, PubMed, Embase, Scopus, Web of Science, SID, Magiran, Irandoc. The search strategy (see Additional file 1 for an example) was first devised for use in PubMed and subsequently adapted for the other databases. The search was limited to papers published between January 1990 and July 2023 and to studies involving economic sanctions independently as a hard power exercise. Other hard power exercises to achieve foreign policy goals such as war and conflicts were excluded. We selected the appropriate keywords from studying similar studies, discussion among research team and intended frameworks for extracting the data. The search term “sanctions” and “public health” were used for PubMed; terms associated with “economic sanctions” and “public health” were used for the title or abstract in the other databases if required (MeSH term; major focus and/or exploded depending on the database). In brief the following terms were searched using Boolean operators: sanction, embargo, health, human resource, medical instrument, medicine, pharmaceutics, disease, mortality, medical equipment, medical devices, drug, health care, Taskforce, health personnel, health workers, morbidity, illness, and food.

### Screening and article selection criteria

Duplicate results were removed after searching the databases using Endnote software version X8. After removing the duplications, a screening of publications, based on titles and abstracts was performed by two researchers independently. In second screening, then, the suspected documents were re-examined by a third person from the research team to decide whether to enter or not.

As the final step of screening, the full texts of the remaining publications were independently assessed for inclusion by pairs of reviewers once more and any potential disagreements were resolved through consensus and if necessary by the third opinion from the research team.

The articles not meeting the below criteria were excluded:

- Articles published in languages other than English and Persian.
- Articles available in preprint servers.
- Articles did not match the question and objectives of the research like those related to the effects of wars and conflict on health.
- Conference abstracts, books, reports and dissertations.
- Records not in line with the quantitative, qualitative and mixed-method original articles including letter to Editor, commentary, opinion/viewpoint/perspective.
- Articles published before 1990.

After reaching the final list of studies to be reviewed thoroughly, we supplemented our database search by screening bibliographic of chosen articles to identify any additional relevant publications. The bibliographic of other relevant systematic articles were also searched actively for retrieving other missing articles.

### Data extraction

After finalizing the final list of articles, the full text of the selected articles were studied precisely and required information was extracted. In order to capture the maximum available evidence regarding the effects of economic sanctions, no quality assessment was employed in our systematic literature review. This approach allowed us to include a wide range of studies, regardless of their methodological quality, thus providing a comprehensive overview of the existing literature. This method is consistent with approaches used in narrative synthesis where the primary aim is to summarize broad evidence on a topic rather than critically appraise each study’s quality. The extracted information was divided into two sections. The first one, consisting the bibliographic information included the title of article, the year of publication, the first author, and the title of the journal and the second section reports the frequency of articles according to the main topics addressed in their results.

### Data analysis and presenting results

For identifying key concepts and main themes, each of selected articles studied carefully. After completing the data extraction table, the researchers shared the concepts with other members of the research team, and agreement was reached. As many other factors outside the borders of health system affects the health, generally known as social determinants of health (SDH), we applied two common popular frameworks to categorize the direct and indirect effects impacts of sanctions on health system and public health. To address the direct impact of sanctions on health, Health System Building Blocks framework proposed by World Health Organization (WHO) was proposed which consists of six key components including “service delivery”, “health workforce”, “health information systems”, “access to essential medicines”, “financing” and “government/ leadership”. This framework also covers intermediate (e.g. access, coverage, quality and safety) and four final goals including Improved health (level and equity), Responsiveness, Social and financial risk protection, and Improved efficiency [21, 22].

To cover other effects of sanctions occurring in other sections beyond the health system but affecting health indirectly, the approach of “Social Determinants of Health” was applied which comprises of the following 10 elements, “The social gradient”, “Stress”, “Early life conditions”, “Social exclusion”, “Work”, “Unemployment”, “Social support”, “Addiction”, “Food”, and “Transport“ [23].

## Results

### Search process

A total number of 18,219 articles were identified, which after removing the overlaps, 12,838 articles remained. Following the initial review of the title and abstract of all retrieved articles, a further 12,439 articles were excluded. Out of 399 records, the full text of 390 articles were retrieved and evaluated for eligibility.

**Fig. 1:**
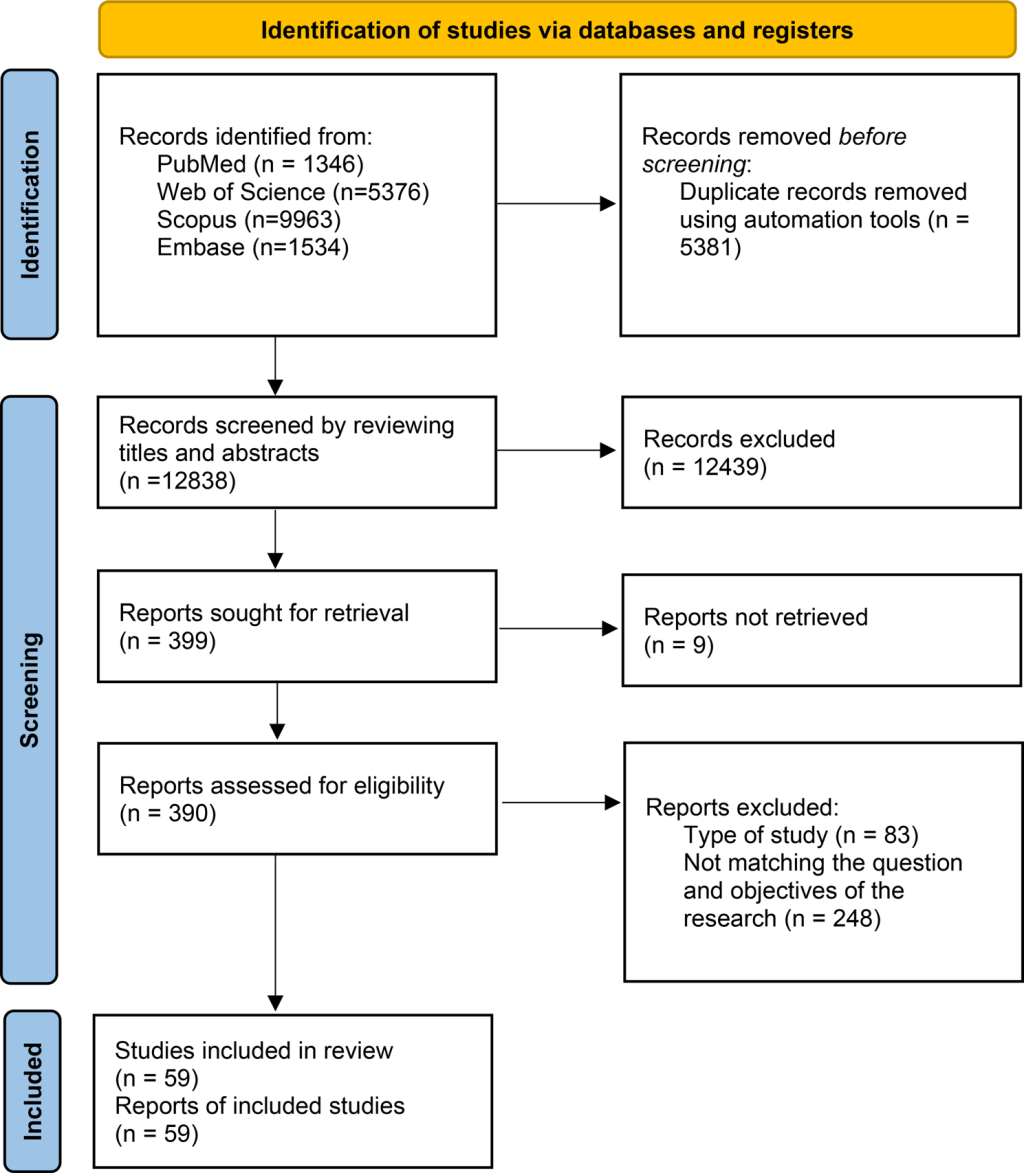
The PRISMA algorithm of study selection process

After a final review, 331 articles were excluded due to not intended study design or not addressing the question and aims of the current research. Finally 59 research articles were included in the study (Fig. 1). A summary of included studies’ features is reported in Table 1.

**Table 1:** A summary of included studies

No.	The title of article	Publication year	First author	Journal title
1	Effect of the Gulf War on Infant and Child Mortality in Iraq (Ascherio et al., 1992)	1992	Ascherio A.	New England Journal of Medicine
2	The health impact of economic sanctions (Garfield et al., 1995)	1995	Garfield R.	Bulletin of the New York Academy of Medicine: Journal of Urban Health
3	The Impact of the Economic Crisis and the US Embargo on Health in Cuba (Garfield and Santana, 1997)	1997	Garfield R.	American Journal of Public Health
4	Infectious diseases mortality in central Serbia (Vlajinac et al., 1997)	1997	Vlajinac HD.	J Epidemiol Community Health
5	Epidemic optic and peripheral neuropathy in Cuba: A unique geopolitical public health problem (Hedges III et al., 1997)	1997	Hedges III TR.	Survey of Ophthalmology
6	The impact of economic sanctions on health and human rights in Haiti, 1991–1994 (Gibbons and Garfield, 1999)	1999	Gibbons E.	American Journal of Public Health
7	The effect of economic sanctions on the mortality of Iraqi children prior to the 1991 Persian Gulf War (Daponte and Garfield, 2000)	2000	Daponte BO.	American Journal of Public Health
8	Sanctions and childhood mortality in Iraq (Ali and Shah, 2000)	2000	Ali MM.	The Lancet
9	A multivariate method for estimating mortality rates among children under 5 years from health and social indicators in Iraq (Garfield and Leu, 2000)	2000	Garfield R.	International Journal of Epidemiology
10	Healthcare under sanctions in Iraq: an elective experience (Akunjee and Ali, 2002)	2002	Akunjee M.	Medicine, conflict, and survival
11	Hapatitis B infection among Iraqi children: The impact of sanctions (Ali, 2004)	2004	Ali H.	Eastern Mediterranean Health Journal
12	Long Term Follow-Up of Renal Transplant Patients-a Single Center Experience in Iraq (Al-Taee and Al-Shamaa, 2005)	2005	Al-Taee IKS.	Saudi J Kidney Dis Transplant
13	Economic crisis and access to care: Cuba’s health care system since the collapse of the Soviet Union (Nayeri and López-Pardo, 2005)	2005	Nayeri K.	International Journal of Health Services
14	Snake bites in north east Sri Lanka (Whitehall, 2007)	2007	Whitehall JS.	Rural and remote health
15	The effect of an international embargo on malnutrition and childhood mortality in rural Haiti (Reid et al., 2007)	2007	Reid BC.	International Journal of Health Services
16	Preconception Health Status of Iraqi Women After Trade Embargo (Abbas et al., 2008)	2008	Abbas WAK.	Public Health Nursing
17	The Impact of War and Economic Sanction on the Incidence of Retinopathy of Prematurity in Serbia (Mladenovich and Langeggen, 2009)	2009	Mladenovich D.	Journal of Visual Impairment & Blindness
18	Causes and differentials of childhood mortality in Iraq (Awqati et al., 2009)	2009	Awqati NA.	BMC Pediatrics
19	Economic Sanctions and Human Security: The Public Health Effect of Economic Sanctions (Peksen, 2011)	2011	Peksen, D.	Foreign Policy Analysis
20	Secular trend of infant mortality rate during wars and sanctions in Western Iraq (Al-Ani et al., 2011)	2011	Al-Ani, Z. R.	Saudi Medical Journal
21	Childhood atopic diseases and early life circumstances: An ecological study in Cuba (van der Werff et al., 2012)	2012	van der Werff, S. D	PLoS ONE
22	Public health services, an essential determinant of health during crisis. Lessons from Cuba, 1989–2000 (De Vos et al., 2012)	2012	Pol De Vos	Tropical Medicine & International Health
23	Economic sanctions: A blunt instrument? (Allen and Lektzian, 2013)	2013	Allen, S. H.	Journal of Peace Research
24	Syria: Effects of conflict and sanctions on public health (Sen et al., 2013)	2013	Kasturi Sen	Journal of Public Health (United Kingdom)
25	Humanitarian impacts of economic sanctions on Iran and Syria (Moret, 2015)	2015	Erica S. Moret	European Security
26	The impact of international economic sanctions on Iranian cancer healthcare (Shahabi et al., 2015)	2015	Shohreh Shahabi	Health Policy
27	The effects of economic sanctions on disease specific clinical outcomes of patients with thalassemia and hemophilia in Iran (Karimi and Haghpanah, 2015)	2015	Karimi, M.	Health Policy
28	A news media analysis of economic sanction effects on access to medicine in Iran (Kheirandish et al., 2015a)	2015	Kheirandish, M.	Journal of Research in Pharmacy Practice
29	Economic Sanctions Against Iran, and Drug Use in Tehran, Iran: A 2013 Pilot Study (Deilamizade and Esmizade, 2015)	2015	Abbas Deilamizade	Substance Use & Misuse
30	Drug adherence of patients with epilepsy in Iran: the effects of the international economic sanctions (Asadi-Pooya et al., 2016)	2016	Ali A. Asadi-Pooya	Acta Neurologica Belgica
31	The Impact of the Sanctions Made Against Iran on Availability to Asthma Medicines in Tehran (Ghiasi et al., 2016)	2016	Golbarg Ghiasi	Iranian Journal of Pharmaceutical Research
32	Addressing the impact of economic sanctions on Iranian drug shortages in the joint comprehensive plan of action: promoting access to medicines and health diplomacy (Setayesh and Mackey, 2016)	2016	Sogol Setayesh	Globalization and Health
33	The Impact of Economic Sanctions on Income Inequality of Target States (Afesorgbor and Mahadevan, 2016)	2016	Afesorgbor SK	World Development
34	Epidemiology and outcome of 2,590 burned patients in northwest Iran (Hosseini et al., 2017)	2017	Hosseini S.N	Annals of Burns and Fire Disasters
35	Analyzing the views of researchers at the Razi Vaccine and Serum Research Institute on the effect of foreign sanctions on scientific communication and research activities (Almasi et al., 2016)	2017	Kobra Almasi	The Scientometrics Research Journal (Scientific Bi-Quarterly of Shahed University)
36	Does Economic Instability Affect Healthcare Provision? Evidence Based on the Urban Family Physician Program in Iran (Rad et al., 2017)	2017	Rad, E. H.	Korean Journal of Family Medicine
37	Current and Future Challenges of Radiation Oncology in Iran: A Report from the Iranian Society of Clinical Oncology(Ameri et al., 2018)	2017	A. Ameri	Clinical Oncology
38	The impact of war and economic sanctions on the mental health system in Iraq from 1990 to 2003: a preliminary report (Younis and Aswad, 2018)	2018	Younis, M. S.	Intervention-International Journal of Mental Health Psychosocial Work and Counselling in Areas of Armed Conflict
39	(Dis)connectivities in wartime: The therapeutic geographies of Iraqi healthcare–seeking in Lebanon (Dewachi et al., 2018)	2018	Omar Dewachi	Global Public Health
40	Impact of economic sanctions on access to noncommunicable diseases medicines in the Islamic republic of Iran (Kheirandish et al., 2018)	2018	Mehrnaz Kheirandish	Eastern Mediterranean Health Journal
41	Impacts of the international economic sanctions on Iranian patients with epilepsy (Asadi-Pooya et al., 2019)	2019	Ali A. Asadi-Pooya	Epilepsy & Behavior
42	Do economic sanctions affect protectionism? Evidence from agricultural support (Lv and Xu, 2019)	2019	Zhike Lv	Economics & Politics
43	Economic sanctions and child HIV (Kim, 2019)	2019	Kim, Y.	Int J Health Plann Manage
44	Impact of United States political sanctions on international collaborations and research in Iran (Kokabisaghi et al., 2019)	2019	Fatemeh Kokabisaghi	BMJ Global Health
45	Economic sanctions threaten population health: the case of Iran (Aloosh et al., 2019)	2019	M. Aloosh	Public Health
46	Economic sanctions and academia: Overlooked impact and long-term consequences(Bezuidenhout et al., 2019)	2019	Louise Bezuidenhout	PLoS ONE
47	Are UN and US economic sanctions a cause or cure for the environment: empirical evidence from Iran (Fotourehchi, 2020)	2019	Zahra Fotourehchi	Environment, Development and Sustainability
48	Trade Sanctions and Agriculture Support in Milk andDairy Industry: Case of Russia (Krivko and Smutka, 2020)	2020	Mikhail Krivko	Sustainability
49	Physical rehabilitation in Iran after international sanctions: explored findings from a qualitative study (Shahabi et al., 2020)	2020	Saeed Shahabi	Globalization and Health
50	Iran, sanctions, and the COVID-19 crisis (Abdoli, 2020)	2020	Amir Abdoli	Journal of Medical Economics
51	Sanctioned to Starve? The impact of economic sanctions on food security in targeted states (Afesorgbor, 2021)	2020	Sylvanus Kwaku Afesorgbor	Research Handbook on Economic Sanctions, 2021
52	Economic sanctions against Iran as an important factor in threatening the health of patients with multiple sclerosis (Sahraian et al., 2021)	2020	Mohammad Ali Sahraian	Current Journal of Neurology
53	The Effect of Sanctions on Iran’s Health System Using Provincial Data and Spatial Panel Methods from 2009 to 2016 (Abhari et al., 2020)	2020	Behroz Abhari	Journal of Health Administration
54	Sanctioned to Death? The Impact of Economic Sanctions on Life Expectancy and its Gender Gap (Gutmann et al., 2021)	2020	JERG GUTMANN	The Journal of Development Studies
55	The impact of smart and non-smart sanctions on government health expenditures: evidence from developing resource-based countries (Faraji Dizaji and Ghadamgahi, 2021)	2021	Sajjad Faraji Dizaji	
56	Relationship between Political-Economic Sanctions and Catastrophic Health Costs in Multiple Sclerosis Patients in Iran (Gharibi et al., 2022)	2022	Gharibi F	Iranian Journal of War & Public Health
57	Challenge of Politico-Economic Sanctions on Pharmaceutical Procurement in Iran: A Qualitative Study (Bastani et al., 2022)	2022	Peivand Bastani	Iranian Journal of Medical Sciences
58	An investigation of relationship between global conomic sanction and life expectancy: do financial and institutional system matter? (Ha and Nam, 2022)	2022	Le Thanh Ha	Development Studies Research
59	The Effects of the Re-imposition of US Sanctions on Food Security in Iran (Hejazi and Emamgholipour, 2022)		Jalal Hejazi	International Journal of Health Policy and Management

### Study features

**Fig. 2:**
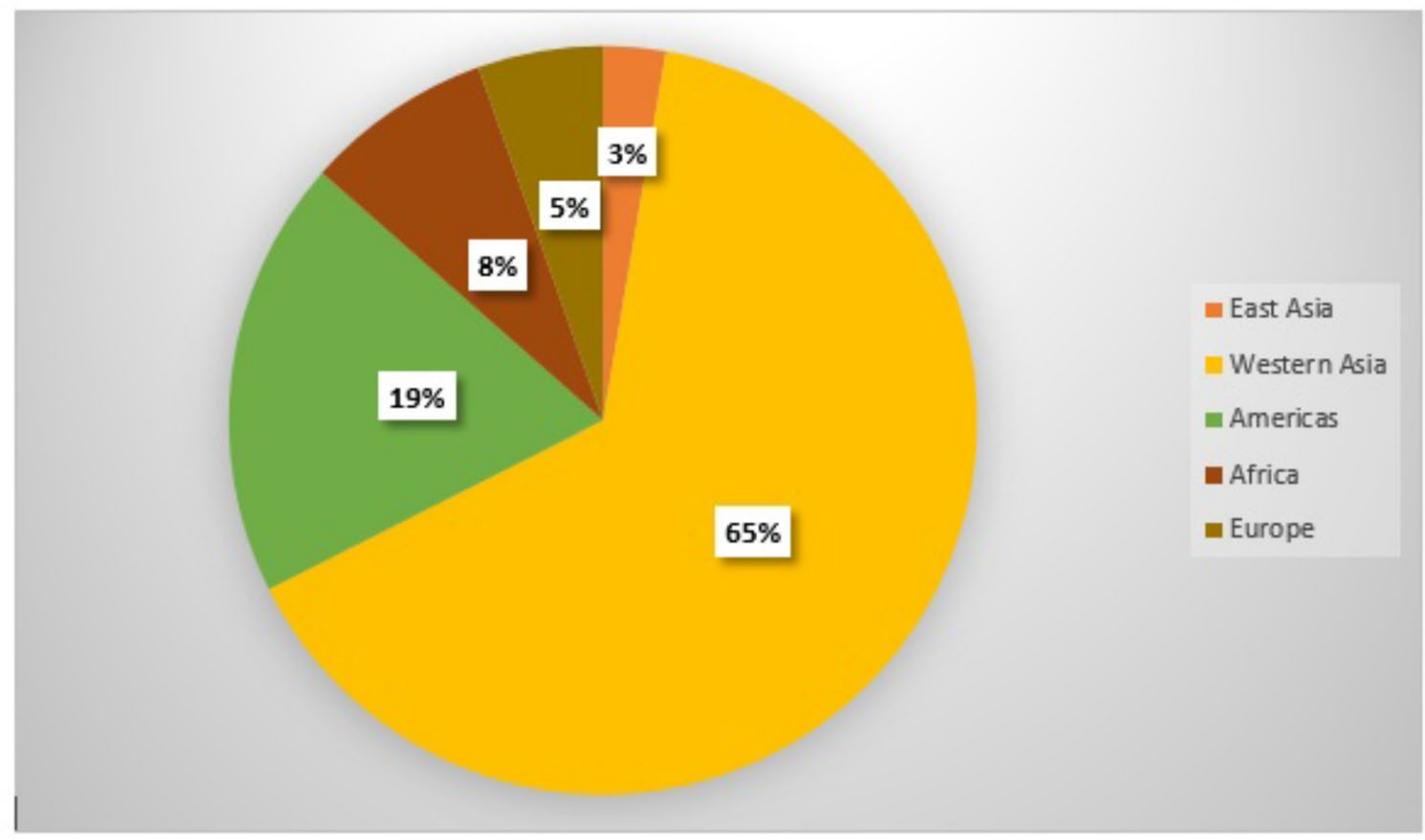
The different regions share of economic sanctions studies

The study information collected from 11 countries which included Iran, Iraq, Cuba, Syria, Haiti, Yugoslavia, Lebanon, Serbia, Nicaragua, Sri Lanka, Russia and South Africa. Iraq [4, 24–35] and Iran [36–45] had the maximum number of studies. Most of the studies were descriptive or analytical. Four were qualitative and also three studies were designed as a mix-method. The share of different regions from economic sanctions studies is shown in Fig. 2.

### Study areas

Included studies have examined the impact of sanctions in various areas. The amount of available and accessible information about the effect of sanctions varied from one area to another. Most studies addressed the impact of economic sanctions on access to medicine or food and also socioeconomic status.

**Fig. 3:**
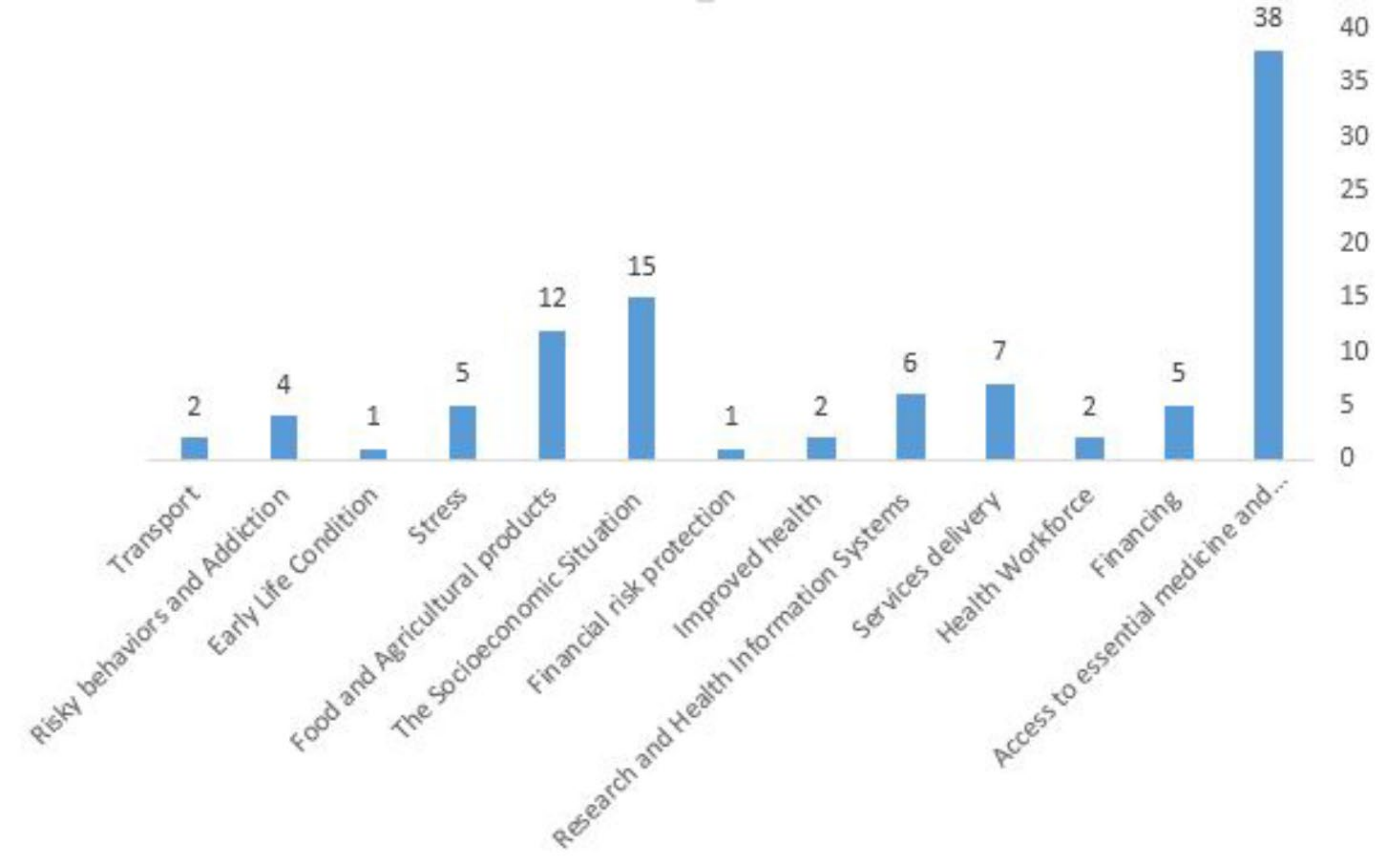
The proportion of different parts of health system and social determinants of health mentioned in retrieved studies which are affected by economic sanctions

Almost half of studies mentioned the effect of sanctions on access to drugs, these studies covered approximately all countries targeted by sanctions. More than a quarter, discussed the socioeconomic situation. Food access and malnutrition were also explained in about another quarter of the articles. According to studies, the vulnerable groups which affected most by sanctions were the poor, patients, women and children. The proportion of different parts of health system and different social health determinants affected by economic sanctions is reported in Table 2; Fig. 3.

**Table 2:** The frequency of direct and indirect effects of sanctions on health mentioned in the selected articles

Scope	Theme	Frequency (%)
A) Direct effects of sanctions on health system	Access to essential medicine, medical products, vaccines, and technologies	22 (37.3)
	Financing	3 (5.1)
	Health workforce	1 (1.7)
	Services delivery	4 (6.7)
	Research and health Information systems	3 (5.1)
	Improved health	1 (1.7)
	Financial risk protection	1 (1.7)
B) Indirect effects of sanctions on population/public health	Socioeconomic situation	9 (15.2)
	Food and agricultural products	7 (11.9)
	Stress	3 (5.1)
	Early life condition	1 (1.7)
	Risky behaviors and addiction	2 (3.4)
	Transport	2 (3.4)
***Total***		***59 (100.0)***

The Table 2 provides an overview of the frequency of direct and indirect effects of sanctions on health, based on findings from 59 selected articles. The data is categorized into direct effects on the health system and indirect effects on population/public health, highlighting both the immediate and broader consequences of sanctions. The most frequently mentioned direct effect was the impact on access to essential medicine, medical products, vaccines, and technologies, with 22 documents, accounting for 37.3% of the papers. On the other hand, the most frequently cited indirect effect was on the socioeconomic situation, mentioned in 9 documents (15.2%).

### Themes and sub-themes

The effects of sanctions on health were categorized into two broad direct and indirect groups. Following the WHO’ Health System Building Blocks, direct effects include 7 main themes as followed: access to essential medicine, medical products, vaccines and technologies; financing; health workforce; service delivery; research and health information systems; health outcomes; and financial risk protection (Table 3). Indirect effects also were summarized in 6 main themes consisting: socioeconomic status; food and agricultural products; stress; early life conditions; high-risk behaviors and addiction; and transportation (Table 4).

**Table 3:** Direct impact of sanctions on health categorized in health system building blocks framework

Theme	Sub-theme	Code
Access to essential medicine, medical products, vaccines, and technologies	Reduced access to imported raw materials	Significant reduction in imported raw materials for producing asthma medications
		Reduced access to imported essential drugs
		Lack of raw materials for making local drugs for cancer, diabetes and heart disease
		Reduction of laboratory services and chemical materials
	Reduced access to drugs	Decreased access to imported asthma medications
		Shortage of 73 drugs, 44% of these drugs classified as essential medicines
		Decreased access to or unavailable chronic patients’ drugs
		Decreased access to or unavailable chemotherapy drugs
		Decreased access to or unavailable psychiatric drugs
		Decreased access to or unavailable hemophilia and thalassemia drugs
		Decreased access to or unavailable MS drugs
		Decreased access to or unavailable cancer drugs
		Decreased access to or unavailable antiepileptic drugs
		Decreased access to burn medications
		Reverse trafficking drugs due to an imbalance between the exchange rate inside and outside the country
		Lack of choice by patients
		Concerning of MS patients about unavailability of internationally branded medicine
		Concerning of MS patients about replacing their medicines with cheaper alternatives due to financial problems
	Increased drug prices	Increased drug prices
		Sharp rise in the price of locally produced medicines
	Decreased rate of imported medical equipment	Reduced X-Rays
		Decreased available medical goods and equipment
		Rising dependence on smuggled goods
		Shortage of raw materials for producing the orthoses and prostheses
		Decreased hospital machinery, and sterilising machines
	Decreased available medical equipment	Decreased available surgical equipment
		Decreased hospital beds
		Malfunction of brachytherapy centers
	Reduced access to bioproducts	Reduced access to vaccines
		Reduced access to drinking water
		The reduction of country’s ability to produce chlorine
		Reduced chlorination
Financing	Financing crisis	Reduced financial support of health services
	Fund health services	Budget limitation
		Reducing governmental public health expenditures
Health workforce	Reduction rate of health workforce	Widespread layoffs of healthcare professionals
	Labor outflow	Physician Immigration
Health services delivery	Reduction rate of provided services	Reduction of performed surgeries
		Reduction of provided health care
		Reduced quality of healthcare
		Reduction of laboratory and surgical services
		Increased gap between Iran’s available facilities for radiation therapy and international standards
Research and health information systems	Reduced access to scientific resources and virtual sites	Reduced access to magazines, textbooks
		Banking restrictions complicate research-related purposes
		Affecting access to international research funds and to form and sustain international collaborations
		Lack of access to software and lack of access to software updates
		Reduced access to internet sites
		Reduced access to essential medical and laboratory supplies
	Disruption of international interactions and conferences	Reduced scientific exchanges
		The limited access to education materials
		Limited educational travels
	Limited research activities	Decreased quality and quantity of research activities
Improved health	Worse health outcomes	Reduced life expectancy especially vulnerable members of society
		Increased child mortality and cholera deaths
Financial risk protection	Worse financial outcomes	Increased out-of-pocket and catastrophic health expenditures, and disease related poverty
		Worsening income inequality

**Table 4:** Indirect effects of sanctions on health categorized in the European SDH agent framework

Theme	Sub-theme	Code
Socioeconomic situation	Increased unemployment	Unemployment related to declining export products
		Reduction of occupied workers in factories
	Decreased income	Reduction in gross domestic production growth
	Reduced welfare	Inflation, increasing living costs, and depreciation of Iranian currency
		General decline of socio-economic well-being
	Increased poverty	Sale of houses and apartments in middle-class due to poverty
		Reduced rate of enrolled poor children in Schools
	Trade barriers	Reduced rate of investment
		Loss of markets, credits and trade conditions
		Devaluation of the national currency against the dollar
		The oil exports stoppage
		Reduced imports of basic goods
		Insurance bans, and increased transport costs
		Facing difficulties in finding third-country banks to process transactions
	Increased prices and decreased purchasing power	Increased rate of household’s basic costs
		Increased prices of imported products
		Increased heating oil prices (increased rate of respiratory patients due to the cold weather)
		Decreased purchasing power of essential foods and medicines
		Rise in commodities and energy costs
		Blocking globalization and not creating political or social change quickly
Food and agricultural products	Increased food prices	Increased prices of main foods
		Increased prices of basic goods like wheat
		Significant increase in the prices of all food groups especially in vegetable, meat, and fruit groups
	Restrictions on proper diet	Lack of B vitamins foods
		Increased malnutrition in pregnant women
		Dietary rations and reduction of supplements for infants
		Reduced family meals
		Reduced protein and calorie intake per capita
		Reduced food consumption
		Increased malnutrition
		Making it difficult to follow a healthy diet
	Food security	Reduced availability and stability of food
		Development of local dairy and milk sector and increasing of its efficiency.
		Shifting from state support of agricultural producers to market price support
	Agriculture	Decreased production or available agricultural products
		Reduced agricultural support
Stress	Increased stress	Fear and uncertainty
		Decreased life expectancy
		Increased mental health problems
Early life condition	Increased adverse effects on long-term health outcomes	Increased percentage of underweight children under five years
Risky behaviors and addiction	Rising risky behaviors	Increased rate of suicide
		Increased rate of violence
		Increase in rates of domestic violence
	Addiction	Common use of syringes for drug injection
		Increased number of drug trafficking networks
		Increased illegal methods of earning money for compensation of drug abuse costs
		Increased drug prices and declined quality of life for abusers
Transport	Worse environmental outcomes	Damaging the target of CO2 emissions

### Direct effects

#### Access to medicines, medical products, vaccines, and technologies

Access to medicine is one of the main goals of health systems. Numerous studies have been reported on drug shortages and public concerns about patients’ difficulties for getting their essential’s [46]. The findings related to access to medicine were divided further into three sub-themes: reduced access to imported raw materials, decreased access to imported or foreign drugs, and increased drug prices.

There are some findings indicated that sanctions prevent the import of essential medical supplies [29, 36, 44, 47, 48]. Therefore, the manufacture of local drugs is affected and access to them is reduced.

For example, Iran experienced a significant decrease in access to asthma drugs which produced locally in Iran, because of local producers relied on imported raw materials [36]. In Yugoslavia, as a result of imposing restrictions on pharmaceutical industry, the available essential drugs decreased by more than 50% [47]. Syria also faced a shortage of raw materials for producing domestic drugs for heart disease, cancer and diabetes [48]. In a similar way, in Iraq, the provision of laboratory services reduced because of raw chemicals shortage [29].

Limited access to imported drugs was another direct effect [49]. The shortage of essential medicines in countries suffering from sanctions was a main concern and access to such medicines including chemotherapy, chronically illness treatments, psychiatric services, MS and antiepileptic drugs was limited considerably [29, 31, 36, 37, 40, 42, 44, 47, 50]. Access to hemophilia and thalassemia drugs was severely affected too [39]. Problems caused by economic sanctions also affected the pharmaceutical market which as a result, lead to an sharp increase in the prices [5, 42, 51].

Studies showed that economic sanctions reduced the import of and access to medical equipment to great extent [3, 24, 29, 52]. In Cuba, the number of X-rays decreased by 75% [3]. Many American companies refused to sell drugs or medical equipment assigned for Nicaragua. Severe shortage of medical products in health system became apparent in 1985 and worsened in 1986 [24].

Also, studies revealed that economic sanctions have reduced access to vaccines and caused less immunization against infectious diseases [25, 53].

In case of Cuba, the country’s ability to produce chlorine decreased and the number of populations with no access to safe drinking water increased, therefore population covered by chlorine water systems decreased from 98% in 1988 to 26% in 1994 [3].

### Health financing

Health financing counts as essential ability of health systems to maintain and improve the community well-being. The economic crisis is affecting the financial capacity of health care system and has hampered the financial support for providing health services [52, 54]. During the economic sanctions, budget constraints also prevented some health care programs from being fully implemented [31].

### Health workforce

A study done in Iraq showed that economic sanctions resulted in widespread expulsions of health care professionals, while many of them were belong to foreign nationals. Also, physicians had to do a lot of extra work, along with increasing pressures which caused them.

leave their jobs behind [29, 31].

### Health services delivery

The imposition of economic sanctions, resulted in labor shortages, limited access to medical equipment, affecting the process of providing health services and made it worse. This imposed much more pressure on the ability of health system as whole particularly during crises like the COVID-19 pandemic [55]. Meanwhile various studies showed a reduction in quantity and quality of provided services too [5, 29, 31, 52, 56, 57]. A study in Iran showed that due to economic sanctions, from 18 brachytherapy centers in 2018, only two centers were usable since 2015 and also the gap between Iran’s available facilities for radiation therapy and international standards deepened [58]. According to the Program of Action for Cancer Therapy, sanctions have disrupted Iran’s National Cancer Control Program (NCCP) as they have influenced all phases of treatment from prevention, to diagnosis/treatment, palliative care, monitoring, and also technology and drug availability [59].

### Research and health information systems

The effects of economic sanctions on research and health information systems were divided into three sub-themes: reduced access to scientific resources and virtual sites, disruption of international interactions and conferences, and restricted research activities. Sanctions also limited access to scientific magazines and books [29, 52]. Specialists were unable to get visas and travel abroad to attend international conferences, which reduced scientific exchanges [52].

The severe financial pressure of sanctions intensified restrictions of scientific travels and communications with the outside world led to a lack of access to educational materials and global medical advances [31].

Economic sanctions also had negative effects on both research and science production activities, including smaller scientific communication, difficulties in research processes, and consequently, decline in the quality along with quantity of research and science development activities [14, 45, 60].

### Health outcomes and financing risk protection

International evidence about the UN sanctions indicates that they reduces life expectancy by about 1.2–1.4 years on average. It was also shown that this reduction is much more severe in vulnerable groups of society like women. This lower life expectancy in the studied countries occurred to great extent due to higher child mortality and Cholera deaths and also spending less amount of public budget on health care [61, 62]. Studies from Iran show that multiple sclerosis patients faced higher out-of-pocket payments, catastrophic health expenditures and the poverty index [63]. Similarly, studies from other countries show higher mortality rate from infectious diseases and more difficulties for optic and neuropathic patients [64, 65]. Physical rehabilitation experts in Iran also concern about high price that people with physical problems have to pay for prostheses which in turn have negative consequences for practitioners themselves [66].

### Indirect effects

The effects of economic sanctions are not targeted and they also influence sectors other than health which can affect general health indirectly. These effects can be categorized under a general concept as social determinants of health (SDH). The main SDHs extracted from the retrieved studies are as follows:

### Economic and social status

The main target of economic sanctions is the economy and money flows of countries which had negative consequences for countries’ economy themselves and other related areas [67]. The indirect outcomes in the area of Economic and Social Status were categorized into 6 sub-themes including: rising unemployment, decreasing income, declining welfare, increasing poverty, trade barriers, rising prices and decreasing purchasing power.

Majority of studies in Iran, Iraq, Cuba, Haiti, Syria, South Africa addressed the effects of sanctions on the socioeconomic situation [4, 5, 29, 44, 47, 48, 52, 68]. These sanctions banned and reduced the exports of products which in turn caused unemployment among those who relied on importing such products to make money. Unemployment rose sharply in Haiti with the cessation of mango exports, on which many poor people depended [47]. Also, in this country some of factories such as clothing, sports and assembly, reduced the number of workers, which was accompanied by rising the rate of job loss [5].

Continuing this situation, economic problems became more and more prevalent. In Haiti, many people lost their main source of income [5]. In Iraq, wage fell and there was hardly enough to buy the necessities of daily life [29]. A review of studies during this period revealed that the reason of increasing social problems and the disintegration of many family structures was the fall in incomes [5, 44].

On the other hand, poverty increased as soon as economic problems intensified. Some of the middle classes’ families were forced to sell their houses and apartments [29]. Also, school enrollment declined due to the poverty [5].

Another important effect was trade barriers so that reduced the rate of investment and the number of foreign companies. The number of active American companies in South Africa fell from 267 in 1986 to 104 in 1991 [47]. Loss of markets, credits, and favorable trade conditions, devaluation of the national currency against the US dollar, the oil exports stoppage, alongside the reduction of basic goods imports were among the other effects [29, 47, 48]. On the other hand, prices increased while purchasing power decreased [4, 5, 48, 52].

### Food and agriculture

Numerous studies in Iran, Cuba, Iraq, and Haiti have shown that economic sanctions reduced food imports while increased their prices, and restricted proper diet 41) [3, 5, 24, 29, 47]. In Cuba, food imports decreased by almost 50% from 1989 to 1993 as a result of falling rate of imports while shifting to low-quality protein products which posed serious threats on population’s health. In Haiti, staple food prices increased fivefold from 1991 to 1993 [47]. Likewise, the prices of all food groups increased significantly in Iran in 2018 due to the limitations in international financial exchanges, right after the re-imposition of sanctions. The price increase was higher in vegetable, meat, and fruit groups which made it nearly impossible to follow a healthy diet [69].

Prices of basic commodities such as wheat, rice and sugar rose in Iraq, too [29]. On the other hand, the lack of foods containing B vitamins group in Cuba led to the epidemic of neuropathy [47]. Poor nutrition among pregnant women in Iraq increased anemia [24]. Meal and per capita protein intake decreased [3, 5]. At the same time malnutrition also increased during restrictions [5, 35, 47, 70] and furthermore caused reduction in crop production and agricultural support [5, 71]. Other studies also revealed that availability and stability were the most affected dimensions of food security following imposing economic sanctions [72]. A study about the impact of the UN and US economic sanctions on the environment in Iran found that while these sanctions initially improved Iran’s environment in the short term, they had long-term damaging effects [73].

### Stress

Economic sanctions exacerbate stressful conditions. According to studies, increased fear and uncertainty, and increased mental health problems are among the negative effects of sanctions in this category [5, 31, 48, 53, 74].

### Early life conditions

A good start in life means supporting mothers and young children. A study found the exposure to adverse economic conditions in infancy and early childhood was effective in long-term negative health outcomes [75].

### High-risk and addictive behaviors

People turning to high-risk behaviors and addiction along with their consumption patterns can be affected and intensified by economic and social conditions. According to the findings, the effects of economic sanctions in case of risky and addictive behavior were categorized into two sub-groups consist of increasing high-risk behaviors and addiction.

As evidences revealed, economic sanctions increased suicide and violence. Studies shown that the rate of deaths caused by violence and suicides have increased in Yugoslavia and Cuba during limitation periods [3, 47]. In Haiti, charges against children, criminal conspiracy, robbery, and drug use were much more serious [5] and this happened along with another important result which was changing in drug use patterns and increased drug abuse problems.

The common use of syringes for drug injection has increased, posing a risk to abusers. Due to economic problems, people entered mass drug distribution networks and drug trafficking to make money; tried steal or other illegal ways to earn money for buying or supplying drugs. Rising drug prices have led to the neglect family economic basket and reduced attention to the factors such as education and health care, which have resulted in low quality of life for consumers and their families [41].

## Discussion

According to the studies, economic sanctions in different countries affected communities’ health in different ways. Economic sanctions are supposed to force a country’s government to reconsider its policies by putting and imposing economic pressure. Although they should not target humanitarian goods, studies in various countries have revealed their direct and indirect effects on community’s health and threats for people’s right to health. The most important effects were found in the access to medicine and change in socio-economic conditions, while ensuring access to medicines for people who needed them, is one of the most emphasized goals of health systems all around the world [76]. Clearly economic sanctions suppress economic growth of the targeted countries in different ways and the lower economic situation can influence all aspects of the whole community and people’ life including their health status directly and indirectly. The World Bank data confirm that sanctions reduced Iran’s economic growth by 38% within three years, as GDP per capita dropped from US$ 7833 in 2012 to US$ 4862 in 2015. Moreover, unemployment increased from 10.4% in 2013 to 13.1% in 2017, and the economic inequality in household expenditure, measured by Gini coefficient, increased from 37 to 41%, since 2012, due to economic sanction. Clearly this economic inequality can lead to health inequity in population [77]. When the economic situation worsens in general, the financial capacity of health system and also the financial power of people will be affected. Evidence from different studies proved that the general budget of health decreased and out-of-pocket payments increased especially for those patients who depend on foreign and imported drugs [54, 63].

In the present study, surveys in different countries showed that economic sanctions through devaluing the national currency, affected access to health goods and services, including drugs and medical equipment. Countries depending on drug technology, requiring the imported raw materials, experienced a severe restriction for accessing to medicine and drugs. On the other hand, these limitations caused a sudden increase or inflation in the prices of medicine and equipment. This effect would be worse for people who suffer from chronic diseases and are unable to purchase or use health care services [16]. For example, sanctions in 2011 caused a 14 times increase in the price of formula in Iran for infants suffering from food allergies. Besides that, uncertainty about the availability of drugs following sanctions also changes the behavior of people as the stored formula for infants not needing a specific formula which was enough for 2 months was distributed only within 4 days in September, 2018 [78].

This is while the economic sanctions reducing the power of supporting health services by limiting budgets and funds. Health financing is essential for the ability of health systems to maintain and improve human health through keeping them capable to fund and provide health services. Without the necessary funding, no health workers will be hired, no medication will be available, and as the same way, no health promotion or prevention will take place [23]. As a result, considering the negative impact of sanctions, the financing system faces serious problems in three main functions of resource collection, pooling, and purchasing. The ability of countries to achieve health system’s goals largely depends on the knowledge, skills, motivation and deployment of individuals to organize and provide health services [23]. Numerous studies showed evidence of a direct relationship between health human resource and population outcomes [79, 80].

Sanctions made it impossible to strengthen service delivery for achieving the Millennium Development Goals (MDGs) related to health by reducing the rate of occupied health workforce and forcing them to migrate. Studies assessed the impact of economic crises shown that these restrictions affected the health sector by increasing public vulnerability as same as the inability to meet public needs and expectations due to limited resources [81–83]. The findings from Iran show that sanctions can influence health care delivery adversely during health pandemics like COVID-19 disease in various direct and indirect ways [55]. Therefore, if sanctions continue, the reduction of inputs will hinder the improvement of service delivery and access to them. On the other hand, they disrupt access to health services even though with minimum quality standards.

About the research, sanctions limited and disqualified research activities by banning the access to most scientific and valid resources and disrupting international interactions and conferences. The small and isolated scientific communication has slowed down the health promotion progresses and limited the access to standards and protocols for promoting public health based on scientific evidence [31, 45].

At the same time, economic sanctions affected on social determinants of health in various dimensions. Loss of markets, credits and favorable trading conditions reduces the value of the national currency and the ability to import goods. While, the cost of basic goods is strongly affected and prices increased [47, 48]. Thus, sanctions forced severe negative effects on people’s health status by reduction of income, welfare, along with increasing unemployment and poverty [5, 29, 84]. This negative impact is more evident on the poor. These people cannot access or buy high or even sometimes low quality health care services.

Another effect of sanctions is reducing access to the food. Sanctions will restrict access to enough food for countries which import their agricultural products. Countries producing their own food products have better resilience. Limitation on access to basic material and food, as well as the economic pressures and declining incomes, affect the pattern of food consumption. Increasing the price of all kinds of food, turning to poor quality and unhealthy foods, as well as buying cheaper, low-nutrient ones, exacerbate malnutrition and make following a healthy diet impossible [69, 70, 85]. It should be noted that imposing sanctions are not always bad and sometimes they force countries to redesign their internal processes. The experience of Russian shows that although sanctions adversely influence agriculture to some extent but instead they played as the new momentum and help its dairy and milk sector to devise positive changes and increase the volume of inter-regional trade in milk and dairy products [86].

From a psychological point of view, the poor and fragile economic situation reduces the value of assets as same as the loss of purchasing power, which leads to increased frustration and stress in people. Frustration and despair cause and exacerbate various diseases [87]. Stressful situations also make people feel anxious, worried and unable to cope with. Psychosocial risks accumulate throughout life and increase the likelihood of poor mental health and premature death [23]. Findings from Iran proved that sanctions also affect mental health adversely. According to the WHO’ data, sanctions led to an increase in death due to self-harm and interpersonal violence in Iran (from 5.9 to 6.1 and from an average of 2.0 to 2.7 per 100,000 persons respectively) during the 2011–2014 period. Interestingly the self-harm related death reduced again in 2016, a year after lifting the sanctions [77].

Risky behaviors and addiction are taking as new behaviors patterns as a result of economic hardships and raised prices. Drug abuse has devastating effects on human health, while increase the rates of crime and mortality [88]. The highest incidence rate of HIV is among drug abusers and their sexual partners [89]. Thus, sanctions intensify these behaviors and patterns with a growing trend, which counts as a great threat to society and especially health system. A review of literature provides many evidence of sanctions effects on health while evidence was provided for most affected areas.

Many studies looked at the effects on access to medicines and medical equipment, research and health information systems, the socioeconomic situation, food and agriculture, and provided a clear picture of consequences. Although some others, focused on one specific area, the others discussed about the issues such as government or leadership, early life conditions, social isolation, social support, transportation which were less in number with no complete and clear evidence that needs further investigation. The most studies in our review examined the effects of sanctions using data and their analysis, or the pre- and post-sanctions situation. Therefore, according to our methodology and included studies, the extracted evidence is very valuable and reliable.

Understanding the impact of economic sanctions on health systems and social determinants of health is crucial for policymakers, highlighting the importance of collaborative global health governance. To address these challenges, a comprehensive approach is needed to minimize harm to vulnerable populations and promote a more equitable and resilient global health environment. Policymakers must reevaluate the effectiveness and unintended consequences of sanctions on health systems, prioritizing humanitarian concerns and ensuring that public health is not disproportionately affected. It is essential to explore alternative diplomatic strategies that allow for humanitarian exemptions within sanctions to guarantee the continued access to essential medical supplies for affected populations. Global health diplomacy should be leveraged to advocate for the removal or modification of sanctions hindering progress towards health-related Sustainable Development Goals (SDGs). Dialogue and negotiation should be prioritized to address underlying tensions while safeguarding the health and well-being of impacted communities. Establishing robust monitoring systems to track the impact of sanctions on health outcomes and social determinants is crucial. Strengthening multilateral collaborations and partnerships to address the health effects of sanctions is imperative, with international organizations like the World Health Organization (WHO) and United Nations (UN) playing a pivotal role in promoting global cooperation and finding solutions.

### Strengths and limitations of the study

One of our limitations was the lack of access to the full text of some articles due to their publication time. Other studies may have been published in other languages about sanctions are excluded because of inclusion criteria. Other limitations may include losing articles about the impact of sanctions on various aspects of the health system that have lost their chance to be published due to political reasons.

Although the present study examined the impact of sanctions on the health system based on the Health System Building Blocks framework of the World Health Organization and using the approach of European SDH, it seems that in some areas the effects are not clear and further studies need to be done. However, given that the impact of sanctions varies from one country to another, the study has provided comprehensive evidence of the impacts along with consequences on health. The present evidence provides guide and helps with the adoption of international policies considering the goals of the WHO and the promotion of peace all around the world.

## Conclusion

The results showed that economic sanctions imposed on different countries, directly and indirectly have strong negative impacts on health. Escalation of sanctions will be a severe threat and barrier for achieving the goal of global health coverage for everyone and everywhere. The international communities must work and focus on reducing the negative effects of these restrictions. They must anticipate the human effects and use whatever means are needed to prevent them. Some of these negative effects like disability and death, are irreversible. Therefore, it seems better for decision makers to recommend an international prescriptive to prevent such irreparable effects on the population of target countries before imposing sanctions.

### Electronic supplementary material

Below is the link to the electronic supplementary material.

Supplementary Material 1

## Data Availability

All data generated during the current study would be available from the corresponding author on reasonable request.

## Abbreviations

SDH: Social Determinants of Health
SDGs: Sustainable Development Goals
WHO: World Health Organization
