# Supplementary material for: Direct and indirect effects of economic sanctions on health: a systematic narrative literature review
Source: BMC Public Health. 2024 Aug 17;24:2242. doi: 10.1186/s12889-024-19750-w (PMC11330615; doi:10.1186/s12889-024-19750-w)
Supplement: Supplementary file 1 — Supplementary Material 1 [file 12889_2024_19750_MOESM1_ESM.docx]

Additional file 1: Search strategy

| #1 | embargo[Title/Abstract] |
| --- | --- |
| #2 | embargoes[Title/Abstract] |
| #3 | sanction[Title/Abstract] |
| #4 | sanctions[Title/Abstract] |
| #5 | #1 OR #2 OR #3 OR #4 |
| #6 | "Public Health"[Majr] |
| #7 | "Equipment and Supplies"[Majr] |
| #8 | "medical equipment"[Title/Abstract] |
| #9 | "medical instrument"[Title/Abstract] |
| #10 | #7 OR #8 OR #9 |
| #11 | Biopharmaceutics[Title/Abstract] |
| #12 | "Biopharmaceutics"[Majr] |
| #13 | Pharmaceutic[Title/Abstract] |
| #14 | Pharmaceutics[Title/Abstract] |
| #15 | Medicine[Title/Abstract] |
| #16 | "Medicine"[Majr] |
| #17 | Drug[Title] |
| #18 | #11 OR #12 OR #13 OR #14 OR #15 OR #16 OR #17 |
| #19 | "Delivery of Health Care"[Majr] |
| #20 | "Delivery of Health Care"[Title/Abstract] |
| #21 | Healthcare[Title/Abstract] |
| #22 | "health care"[Title/Abstract] |
| #23 | Health[Title/Abstract] |
| #24 | "Health"[Majr] |
| #25 | #19 OR #20 OR #21 OR #22 OR #23 OR #24 |
| #26 | "Workforce"[Majr] |
| #27 | Workforce[Title/Abstract] |
| #28 | "Human resource"[Title/Abstract] |
| #29 | Taskforce[Title/Abstract] |
| #30 | "Health Personnel"[Majr] |
| #31 | "Healthcare worker"[Title/Abstract] |
| #32 | "Health Personnel"[Title/Abstract] |
| #33 | #26 OR #27 OR #28 OR #29 OR #30 OR #31 OR #32 |
| #34 | Mortality[Title/Abstract] |
| #35 | "Mortality"[Majr] |
| #36 | (Morbidity[Title/Abstract] |
| #37 | "Morbidity"[Majr] |
| #38 | #34 OR #35 OR #36 OR #37 |
| #39 | Disease[Title/Abstract] |
| #40 | "Disease"[Majr] |
| #41 | Illness[Title/Abstract] |
| #42 | "Ill health"[Title/Abstract] |
| #43 | #39 OR #40 OR #41 OR #42 |
| #44 | Food[Title/Abstract] |
| #45 | "Food"[Majr] |
| #46 | #44 OR #45 |
| #47 | #6 OR #10 OR #18 OR #25 OR #33 OR #38 OR #43 OR #46 |
| #48 | #5 AND #47 |
